# Selection of housekeeping genes and demonstration of RNAi in cotton leafhopper, *Amrasca biguttula biguttula* (Ishida)

**DOI:** 10.1371/journal.pone.0191116

**Published:** 2018-01-12

**Authors:** Satnam Singh, Mridula Gupta, Suneet Pandher, Gurmeet Kaur, Pankaj Rathore, Subba Reddy Palli

**Affiliations:** 1 Punjab Agricultural University, Regional Station, Faridkot, Punjab, India; 2 Department of Entomology, University of Kentucky, Lexington, KY, United States of America; Chinese Academy of Agricultural Sciences Institute of Plant Protection, CHINA

## Abstract

*Amrasca biguttula biguttula* (Ishida) commonly known as cotton leafhopper is a severe pest of cotton and okra. Not much is known on this insect at molecular level due to lack of genomic and transcriptomic data. To prepare for functional genomic studies in this insect, we evaluated 15 common housekeeping genes (*Tub*, *B-Tub*, *EF alpha*, *GADPH*, *UbiCF*, *RP13*, *Ubiq*, *G3PD*, *VATPase*, *Actin*, *18s*, *28s*, *TATA*, *ETF*, *SOD and Cytolytic actin*) during different developmental stages and under starvation stress. We selected early (1^st^ and 2^nd^), late (3^rd^ and 4^th^) stage nymphs and adults for identification of stable housekeeping genes using geNorm, NormFinder, BestKeeper and RefFinder software. Based on the different algorithms, *RP13* and *VATPase* are identified as the most suitable reference genes for quantification of gene expression by reverse transcriptase quantitative PCR (RT-qPCR). Based on RefFinder which comprehended the results of three algorithms, *RP13* in adults, Tubulin (*Tub*) in late nymphs, 28S in early nymph and *UbiCF* under starvation stress were identified as the most stable genes. We also developed methods for feeding double-stranded RNA (dsRNA) incorporated in the diet. Feeding dsRNA targeting Snf7, IAP, AQP1, and VATPase caused 56.17–77.12% knockdown of targeted genes compared to control and 16 to 48% mortality of treated insects when compared to control.

## Introduction

Gene expression studies are indispensable for molecular biology research. The knowledge on gene expression helps to better understand its regulation and functions. The availability of huge sequence data in the form of transcriptomes and genomes of several organisms can be utilized to understand the transcription of the gene(s) [1]. However, these studies have become more accurate and robust after the development of gene expression quantitation method, reverse transcriptase quantitative PCR (RT-qPCR). This method is highly sensitive, reproducible and accurate to a level that it can identify even minute variations, which are frequently undetected. The RT-qPCR data are influenced by many factors, which include quality and quantity of the starting material, RNA extraction, cDNA synthesis, and other laboratory procedures. Even pipetting errors and reverse transcription efficiency can influence the Ct values significantly[2,3]. Thus normalization is a prerequisite in gene expression studies as it limits variability by comparing target gene expression with housekeeping genes (HKGs). Normalization is based on the assumption that the expression of the HKGs is stable across various biotic and abiotic stresses and treatments. Recent research indicates that a condition-specific reference gene needs to be identified for accurate measurements of gene expression[4]. Likewise, it is also evident that a single reference gene is not desirable for the wider experimental regime[5,6]. The use of a single reference gene can generate up to a 20-fold error in the expression data [7]. In most of the expression studies, actin is taken as a universal HKG [8] or the HKGs validated for certain systems are being directly applied to other without an appropriate validation of their stability in that particular system. To address this, different software programs have been developed to choose HKGs that are most suited for normalization[7–10]. Thus, it is necessary to choose the most suitable genes for normalization from a panel of candidate genes in a dedicated set of biological samples from a particular organism. Although RT-qPCR has been widely used for detecting gene expression in insects but there is yet no suitable HKG and stable gene quantification system for the cotton leafhopper. In addition, there is no report of RNAi (RNA interference) in this insect. We demonstrated feeding RNAi in this insect for few genes such as Aquaporins (AQPs), inhibitor-of-apoptosis (IAP), VATPases and Snf7. Aquaporins (AQPs) belong to the family of the major intrinsic proteins (MIP), which are integral membrane channel proteins in most living organisms and facilitate mass transfer of water and sometimes other substrates across cell membranes [11–14]. In insects, the inhibitor-of-apoptosis (IAP) proteins are a family of conserved survival factors that determine cell fate during development, stress, tumorigenesis, and infection by regulating the phenomenon of apoptosis [15–19]. VATPases are highly conserved and ubiquitous proton pumps which acidify specific organelles such as lysosomes, endosomes or secretory vesicles in every eukaryotic cell. These are present in the plasma membrane of different animal cell types where they are involved either in membrane energization or pH homeostasis [20–22]. Snf7 functions as a component of the ESCRT (Endosomal Sorting Complex Required for Transport) pathway which plays an essential part in cellular housekeeping by internalization, transport, sorting and lysosomal degradation of transmembrane proteins [23]. It has been shown to be involved in sorting of transmembrane proteins either through recycling to plasma membrane or routed to lysosomal degradation through the endosomal-autophagic pathway in many organisms [24–28].

*Amrasca biguttula biguttula* (Ishida) (Hemiptera: Cicadellidae), commonly known as cotton leafhopper/jassid, is among the most economically important sucking pests of cotton. Leafhopper infested tender leaves become small, crinkled, yellow and the margin of the leaves starts curling downwards. In the case of severe infestation, leaves get a bronze or brick red color which is typical “hopperburn” symptom. The leaves dry up and are shed, and the growth of the plant is retarded. Apart from drying of leaves, the punctures of leafhoppers induce the shortening of the internodes, which contribute to reduced plant vigor and yield [29]. The pest has the potential of causing 25–45% loss in seed cotton yield [30,31]. Besides cotton, it also infests many crops in malvaceous and solanaceous families. The mainstay of leafhopper control shifted to neonicotinoids after the development of resistance against most of the pyrethroids and organophosphates [32]. Bt-cotton seeds treated with neonicotinoids protect against cotton leafhopper till 40–50 days after sowing. However, there have been reports of the leafhoppers showing a high level of resistance up to 5,000-fold to imidacloprid and other neonicotinoid insecticides introduced barely decade ago in central India [33]. Besides, the usage of synthetic insecticides is also associated with a number of environmental issues such as insecticide residues in soil and water, and effects on non-targets, etc. The ill effects of pesticides can only be addressed through eco-friendly methods of pest control, but on contrary Bt-toxins (sole candidates of insect- resistant transgenics) are either not available or less effective against sap-sucking pests such as aphids, leafhoppers and whitefly. The present—day RNAi technology has the potential of developing novel insect control strategies. The method relies on feeding/injecting sequence-specific double-stranded RNA (dsRNA) targeted towards downregulation or knockdown of essential genes for causing mortality. The dsRNA targeting such essential genes can help in developing new generation insect resistant transgenic plants. Ironically, in spite of attaining serious pest status on cotton, the molecular genetics of this insect has remained unexplored with information available only on a single gene (mtCOI-taxonomic importance) in the NCBI database. To initiate molecular studies related to gene expression, RNAi, and other future functional genomics studies, selection of right HKGs is a prerequisite. Hence, the studies first time report the suitable HKGs for different developmental stages and under starvation stress in cotton leafhopper. In addition downregulation of targeted genes associated with different physiological functions through dsRNA feeding has been successfully demonstrated in this insect.

## Materials and methods

### Selection of genes and primer design

The selection of genes for identifying stable reference genes in *Amrasca biguttula biguttula* (Cotton leafhopper) was based on previous studies in other insects [34–36]. As per information available, we chose 15 commonly used reference genes such as *Tub* (Tubulin), *β-Tub* (Beta Tubulin), *EF alpha* (elongation factor alpha), *GADPH* (Glyceraldehyde 3-phosphate dehydrogenase), *UbiCF* (ubiquitin-conjugating factor), *RP13* (ribosomal protein 13), *Ubiq* (ubiquitin), *VATPase* (V-type adenosine triphosphatase), *Actin*, *18s* (ribosomal protein 18), *28s* (ribosomal protein 28), *TATA* (TATA-binding protein), *ETF* (electron transfer flavoprotein), *SOD* (superoxide dismutase) and *Cytolytic actin* as shown in Table 1. The sequences of these genes were retrieved from our transcriptome sequence data (unpublished) followed by a BLASTX search of each sequence in NCBI database for re-confirmation of their annotation. The primers were designed using Primer3 [37] software to amplify 100–150 bp region of the respective gene (Table 1). The sequences of all the selected genes have been submitted to NCBI database and are available with Accessions MF101761-MF101776.

**Table 1:** Annotation of different target genes of cotton leafhopper from RNA sequence data using BlastoGo software and primers used for expression analysis of housekeeping genes.

Gene Symbol	Accession number	Locus description	Homolog locus	Primer Sequence (5’ to 3’)	Identity (%)	E value
Tub	MF101761	tubulin alpha chain	XP_011616108	5’AGCGAAGTCATACCCTTGACAC3’ 5’GGTATGTCGAACACGTCAGATG3’	81	1.80E-99
B-Tub	MF101762	beta-tubulin 4	ALP82110	5’CATTCATAGGCAACACCACTGC3’ 5’ACTCTGCCTCTGTGAACTCCAT3’	98	0
EF	MF101763	elongation factor-1 alpha	AAF29896	5’GACGTGTACAAGATCGGTTGGT3’ 5’GCATCTCCACAGACTTGACCTC3’	75	2.61E-55
UbiCF	MF101764	ubiquitin conjugation factor E4	XP_014246421	5’TGACTGATCCGGTTATCCTACC3’ 5’CTTGAGTTCCTCGTCTGGTTTC3’	67	0
RP13	MF101765	39S ribosomal protein L13	XP_008475259	5’ACCAGAGCCATGGAGAGAAGTT3’ 5’GAGGAATTGGTCTCAGAACAGG3’	84	2.15E-84
G3PD	MF101766	glycerol-3-phosphate dehydrogenasedehydrogenase	XP_011881595	5’CCTGACCAAAGAAGAGATCCAG3’ 5’CTCTCCAAAGTGCTTGAGACCT3’	88	0
CyAct	MF101767	putative cytoplasmic actin A3a1	AAT01072	5’AACACAGTTCTGTCCGGAGGTA3’ 5’CCTCCGATCCATACGGAGTATT3’	100	1.46E-57
UbiQ	MF101768	Ubiquitin C	CAX71215	5’CGATTCGACCATGCCTTACTT3’ 5’GAGATTGACACGCTCCTGAAA3’	60	3.30E-88
VATpase	MF101769	V-type proton ATPase	AIY24627	5’GATCAAGGATGACTGGACTGGT3’ 5’AGACGCAGAGTATGGAGGAATG3’	0	90
Actin	MF101771	actin-like protein 6B	XP_014270187	5’CTCCAGTAAGAGGTGGGATAGT3’ 5’CAGTGACAACAACACTACCATAGA3’	0	91
18s	MF101772	18S rRNA (guanine-N(7))-methyltransferase	XP_015126904	5’CACCAAGAACCAAGTCACCTTC3’ 5’GCTGTGGTTCTGGATTAAGTGG3’	87	3.46E-142
28s	MF101773	28S ribosomal protein S15, mitochondrial	KDR19003	5’GAACGGCTAGCAGAATACAAGG3’ 5’CTTCGAAATCAGCCTCTGACAC3’	65	1.14E-50
TATA	MF101774	TATA box-binding protein-like protein 1 isoform X1	XP_012136986	5’AACAGCGTCTATCTGGTCGTCT3’ 5’GAGTCTTGAAGCCGAGTTTCTG3’	73	2.74E-111
ETF	MF101775	Eukaryotic translation initiation factor 3 subunit B	KDR10090	5’GACTGGCCAGACTCCAATAGTC3’ 5’AACTCCAGGGTACCGTTAGCTC3’	85	0
SOD	MF101776	superoxide dismutase [Cu-Zn]-like isoform X2	XP_014255311	5’GGCAGTTTGTACCAGCCTTATC3’ 5’ATCACAGACCCTTCCACAGAGT3’	75	3.93E-61

### Rearing of insect

*A*. *biguttula* insects were reared on *Gossypium hirsutum* variety Ganganagar ageti in the walk-in environmental chamber at 65–70% RH, 14:10 h light and dark photoperiod and 27± 2°C. The plants of the variety were changed from time to time for continuous fresh food supply to the insect and availability of its culture throughout the study period.

### dsRNA synthesis and feeding

To test RNAi in *A*. *biguttula biguttula*, we chose AQP (aquaporin), IAP, (inhibitor of apoptosis) VATPase and Snf7 (vacuolar sorting protein) genes shown in Table 2. Gene specific primers were designed from the transcriptome sequences using Primer3 open source software using RNA sequences [37]. The dsRNA primers were also designed using Primer3.However, an additional T7 promoter sequence was added to 5’ end of both reverse and forward primers as given in Table 2. These primers were used to amplify the template for dsRNA using cDNA which was synthesized using PrimeScript™ 1st strand cDNA Synthesis Kit (Clontech Takara) as per manufacturer’s protocol. Amplified fragments of genes were purified using nucleospin PCR cleanup (Macherey-Nagel Nucleospin Gel and PCR Cleanup) and used for the synthesis of dsRNA with T7 RiboMAX™ Express RNAi System (Promega) following manufacturer’s protocol. The dsRNA was quantified using BioSpectrometer^®^ basic (Eppendorf). The 500ng/μl of dsAQP, dsIAP, dsVATPase, dsSnf7, and dsGFP was mixed with diet [38] as described in Table 3. Three biological replicates of five insects each were released in a 1.5 ml tube (1/4^th^ cut from the bottom side and covered with muslin cloth) with diet incorporated dsRNA between two layers of stretched parafilm. The insects were observed 48 h post release and the mortality was compared to dsGFP treated insects. Live insects were collected in Tri-Reagent^**®**^ (Sigma-Aldrich), frozen in liquid nitrogen and processed for RNA extraction.

**Table 2:** Description of all candidate gene primers used for RNAi and target gene expression in cotton leafhopper.

Gene	dsRNA Primer sequence (5’ to 3’)	qRT-PCR primers (5’ to 3’)
Snf7	5’TAATACGACTCACTATAGGGGCTTTGGCAGTGGTCTTAGC-3’ 5’TAATACGACTCACTATAGGGTAAAAGAGCGGCAATCCAAG-3’	5’GAGCAGTGGAGAAACGAATGAC3’ 5’ACGGGCGTACACAGGTTTACTT3’
AQP	5’TAATACGACTCACTATAGGGACTGCCAAACATGGATGGAT-3’ 5’TAATACGACTCACTATAGGGGGAGCAGTGATTGAAGGCATA-3’	5’CCAGTACAAGCTCCAATCCAGT3’ 5’GGTGGCTGCATTCAACTACTCT3’
VATPase	5’TAATACGACTCACTATAGGGTGGGTGTCTTACAGTGCTATCG-3’ 5’TAATACGACTCACTATAGGGAGAGCCCAGCACGTACTCTATG-3’	5’GATCAAGGATGACTGGACTGGT3’ 5’AGACGCAGAGTATGGAGGAATG3’
IAP	5’TAATACGACTCACTATAGGGCTCAAGAGAGCACTTCCGTTCT-3’ 5’TAATACGACTCACTATAGGGCCTTGGAGTGCTTCTCTCAGTT-3’	5’CGTGGAAGCCTTTACAGTTAGC3’ 5’GGGTGTTTATGTCCGTTACCAG3’

**Table 3:** Synthetic diet composition for delivery of dsRNA in cotton leafhopper through membrane feeding.

Components	Concentration (mg/10 ml)
L-Cysteine	5.0
Glycine	2.0
Nicotinic Acid	1.0
Sucrose	500.0
K_2_HPO_4_	50.0
ZnCl_2_	0.04
Thiamine HCl	0.25
Vitamin B6	0.25
Becosules capsule powder(Pfizer Limited, USA)	2.0
Green food dye (GanpatiSyn Food Colour, India)	2.0

### RNA extraction and cDNA synthesis

For validation of housekeeping genes, insects were categorized into different developmental stages, i.e., early nymph (I and II), late nymph (III and IV) and adult. Three biological replicates of each developmental stage were separated and pooled, i.e., early nymphs 25–30 individuals per pool, late nymphs 15–20 per pool and adult hopper five insects per pool. For expression under starvation stress, late stage nymphs (III and IV) were starved for four h in empty ventilated boxes, and four individuals each were pooled in three different tubes. The total RNA was isolated from each developmental stage, starved leafhopper and dsRNA fed insects using Tri Reagent^**®**^ (Sigma-Aldrich) as per manufacturer’s protocol. Isolated RNA was given DNase treatment to remove DNA contamination. The RNA was quantified and checked for its quality and quantity on Eppendorf BioSpectrometer^®^ basic. The total RNA (2μg) was used for first-strand cDNA preparation using PrimeScript™ 1st strand cDNA Synthesis Kit (Clontech Takara) as per manufacturer’s protocol.

### RT-qPCR analysis

qPCR analysis used SYBR® Premix Ex Taq™ II (TliRNase H Plus) (Clontech) with the LightCycler®96 (Roche Molecular Systems, Inc.). PCR reactions were performed in triplicates in 10μl volumes using 1μl of 1:10 diluted cDNA, 0.2 ul of 10mM of gene-specific primers per reaction. For absolute quantification, three 10-fold serial dilutions were performed to ensure that the cDNA synthesis reagents did not impair PCR efficiency. Thermal cycling conditions constituted initial denaturation of 95°C for the 30s followed by 40 cycles of 95°C of 5s and 60°C for 10s. Melting curve analysis was done to ensure the specificity and consistency of the amplified product. RT-qPCR efficiency was measured for each gene with the slope of linear regression model and standard curves. Amplification efficiency and correlation coefficients for each primer pair were calculated as described in LightCycler®96 users guide. In RNAi studies, the relative expression of genes in biological samples was estimated using the ΔΔCt method [39] normalized with RP13 as HKG and compared with dsGFP.

### Stability and statistical analysis for reference genes

We chose four algorithms to determine the stability of genes, i.e., geNorm [7], NormFinder [9], Bestkeeper [10] and RefFinder [40]. The qPCR obtained Ct values from the Lightcycler software (Roche) were transferred to MS-excel for the calculation of linear relative values by comparative Ct method (keeping lowest relative quantity for each gene as 1). These linear relative quantities were used as input data for further analysis of gene stability with geNorm and NormFinder. geNorm calculates the expression stability score (M) by averaging the mean pairwise variation of each HKG. Lower ‘M’ value indicates the stability of the gene, so the HKGs showing M value > 0.5 were not considered for further normalization studies. NormFinder calculates expression stability of the genes within a group and between the groups. It also determines standard deviation (SD) through advanced analysis. BestKeeper is also a freely available algorithm that directly utilizes the Ct value obtained from the software. It evaluates standard deviation, p-values, index and correlation coefficient of each gene to elucidate the most stable gene. The gene with a lower SD value could serve as a better reference gene. The final assessment was made on the basis of geometric mean calculated for each gene using RefFinder (http://leonxie.esy.es/RefFinder/?type=reference), a web-based tool which uses all three algorithms along with ΔΔCt method for stability analysis of genes. This web-based tool is user-friendly and allows to evaluate and screen the candidate reference genes directly on the basis of Ct values across the samples efficiently. It makes a comprehensive analysis of data obtained from all the algorithms and ranks the candidate genes in decreasing order of their stability. On the grounds of rankings from each program, an appropriate weight is assigned to each candidate gene, and the geometric mean of their weights is calculated for the net final ranking.

## Results

### Verification of expression of selected genes

Fifteen candidate genes based on their significance in the biological processes were tested for their expression. These included the structure-related genes, Tub, B-Tub, Cytolytic Actin and Actin; ribosomal and protein function genes, EF alpha, 18s, 28s, RP13, TATA, ETF; metabolism-related genes, UbiCF, Ubiq, GAPDH, VATPase and SOD. The initial screening of all targeted genes was done by PCR and the amplified products were checked by agarose gel electrophoresis. These analyses verified that all the targeted genes were expressed in *A*. *biguttula biguttula* (data not shown). In order to calculate the amplification efficiency for all fifteen candidate genes, the three-point standard curve was obtained using the Lightcycler software following the PCR amplification with the known concentration of cDNA template. All genes except Ubiq and CytoActin (78.04% and 62.32% respectively) showed significant amplification efficiency (Table 4). So these two genes were eliminated and not taken into account for further analysis. The correlation coefficient (R^2^) for all genes ranged between 0.92~1.00 (Table 4). Melt-curve analysis was also performed for amplification specificity, and all genes displayed a single peak. Purified qPCR products were sequenced, and the sequences matched 100% with the target sequences.

**Table 4:** Primer sequences and amplicon characteristics of the candidate reference genes.

Gene Symbol	Amplicon Length	Product Tm (°C)	Amplification efficiency E (%)	Correlation Coefficient
Tub	122	83	**112.14**	0.99
B-Tub	141	85	**100.85**	0.99
EF	131	86	**102**	1.00
RP13	133	81	**90.42**	0.99
UbiCF	143	84	129.14	0.99
CyAct	144	86	62.32	0.97
G3PD	115	81	**106.18**	1.00
Ubiq	106	79	78.04	0.92
VATpase	123	85	121.20	1.00
Actin	123	83	**99.59**	0.99
18s	129	81	**81.81**	0.96
28s	142	80	**91.52**	0.99
TATA	119	86	**97.96**	0.96
ETF	123	82	**99.87**	1.00
SOD	134	86.6	116	0.98

### Expression profiles of genes

**Fig 1:**
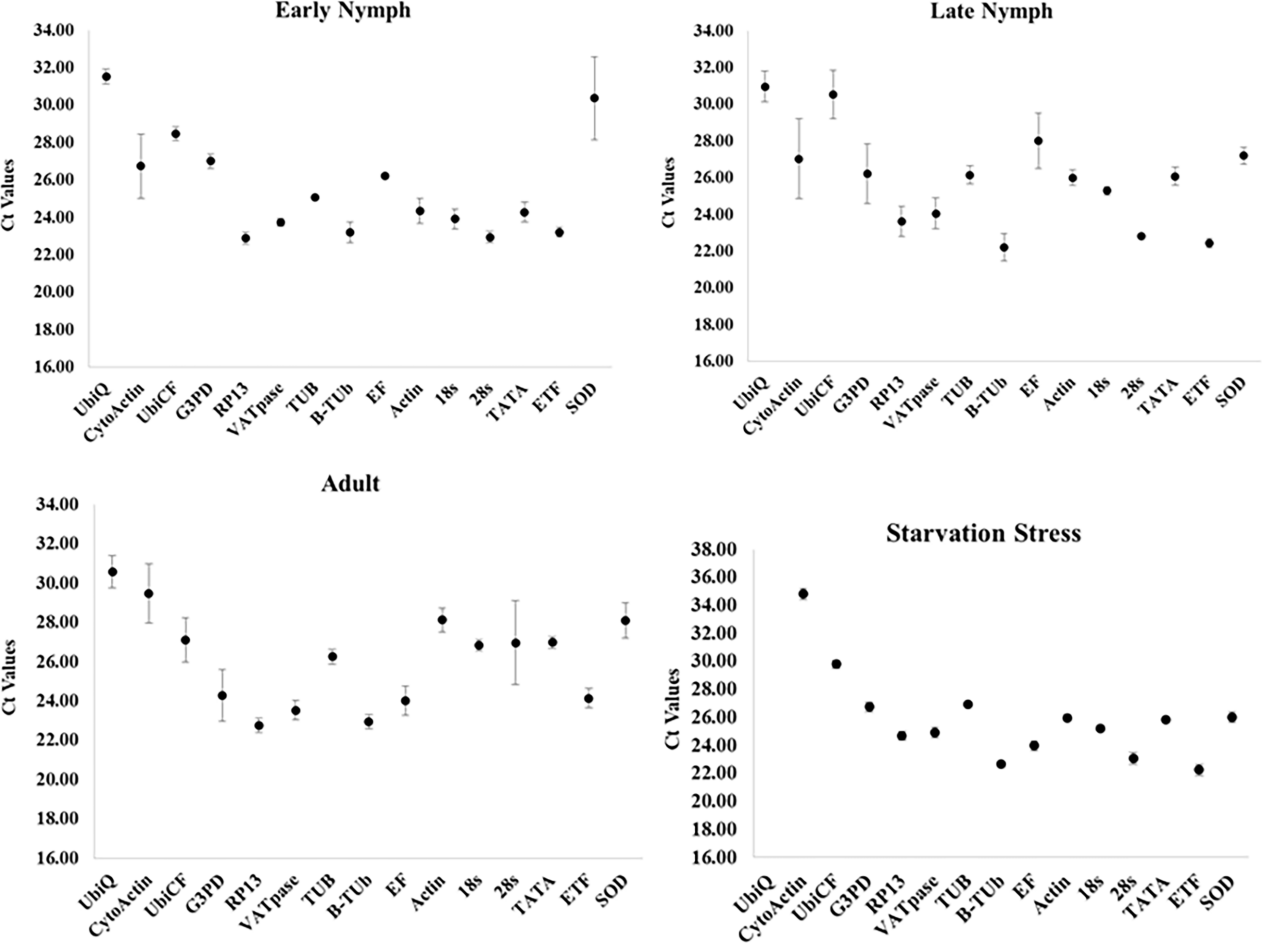
Ct values±S.D. obtained for fifteen candidate reference genes of cotton leafhopper across developmental stages. Each data point represents the Mean±S. D. of Ct values for three biological replications in each treatment.

Relative expression levels were determined using RT-qPCR. The Ct values of the candidate reference genes ranged from 22.3 (B-Tub) to 32.24 (Ubiq). The expression of Ubiq gene was low (29.13~32.50) in all developmental stages tested. In starved individuals, the expression of CytoActin and Ubiq was considerably low, showing higher Ct values as compared to the normal samples. The genes RP13, VATPase and β-Tub, showed Ct values ranging between 22~25 indicating higher expression levels. There was not much difference in Ct values across various stages for each gene except for GADPH, which ranged between 22.69~31.06 (Fig 1).

### BestKeeper analysis

**Fig 2:**
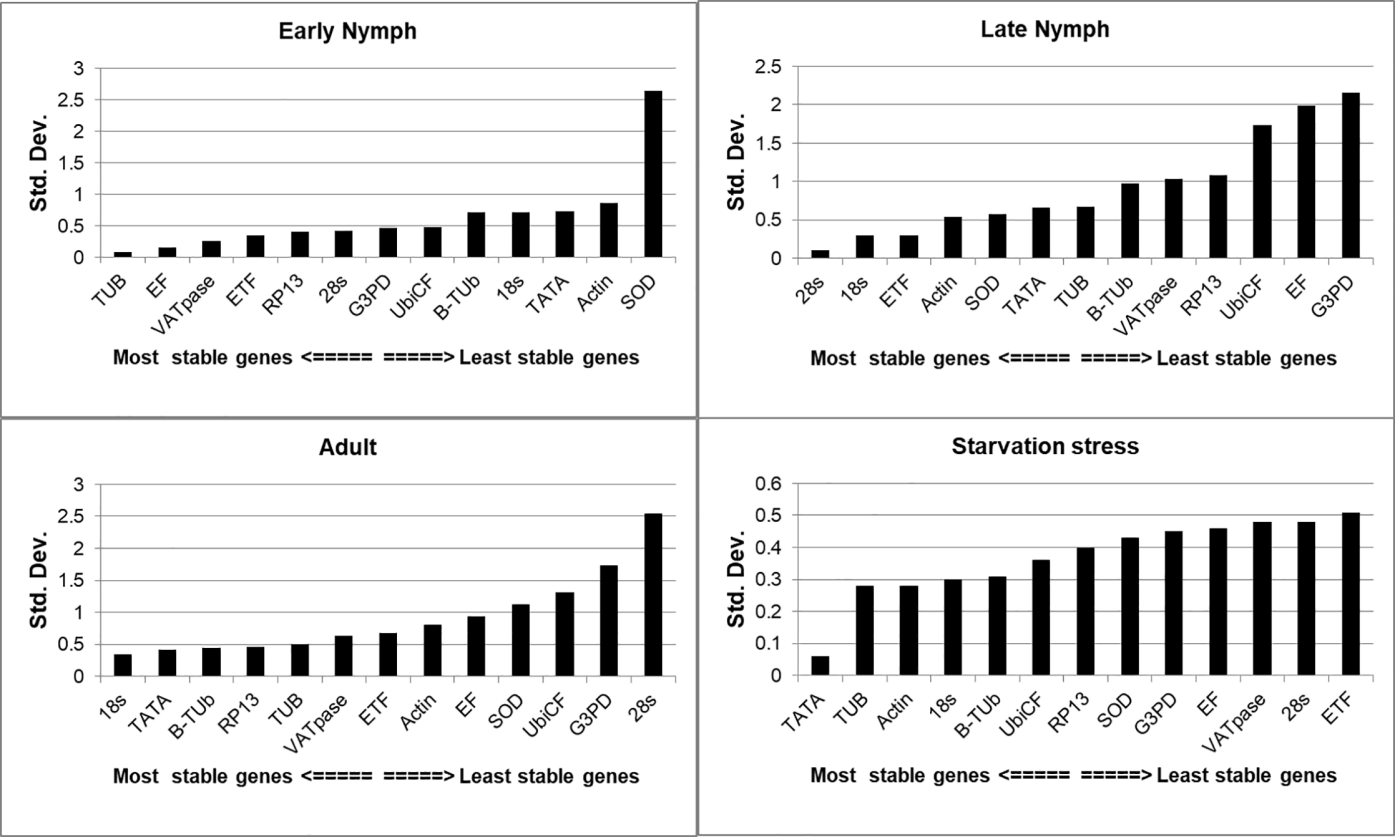
Standard deviation for the Ct values of all genes to determine the stability of the candidate genes using Bestkeeper analysis.

BestKeeper reveals the best genes on the basis of SD value for each candidate reference gene from raw Ct values. An SD > 1 manifests that the diversification in the expression of a gene within a sample of the same origin is high, thus indicating instability in the expression of that gene. Our data signified that expression was not so stable for many genes, i.e., UbiCF, GAPDH, 28s, and SOD in adult samples as it has shown variations across the samples along with high SD values. Similarly, in late nymph, instability was observed in the genes such as UbiCF, GAPDH, RP13, VATPase and EF based on high SD value (Table 5). In adult hopper, 18s was the most stable gene with lowest SD value of 0.34. Similarly, in late nymph 28s (SD = 0.1), early nymph Tub (SD = 0.08) and under starvation stress TATA (SD = 0.06) were the most stable genes. Ranking of all genes in decreasing order of their stability across all the stages is presented in S1 Table and Fig 2.

**Table 5:** Stability values of all candidate reference genes estimated by Normfinder across all developmental stages and starvation stress.

Genes	Adults	Late Instars	Early Instars	Starvation Stress
UbiCF	0.806	3.191	0.217	0.002
G3PD	2.260	2.420	0.377	0.017
RP13	0.009	0.380	0.078	0.087
VATpase	0.009	0.495	0.143	0.066
TUB	1.032	0.000	0.037	0.209
B-Tub	0.576	0.274	0.328	0.002
EF	0.096	4.257	0.062	0.022
Actin	0.276	0.003	0.048	0.123
18s	0.102	0.002	0.026	0.063
28s	4.900	0.173	0.213	0.112
TATA	0.195	0.000	0.025	0.115
ETF	0.347	0.055	0.148	0.049
SOD	0.568	0.048	0.096	0.040

### geNorm analysis

**Fig 3:**
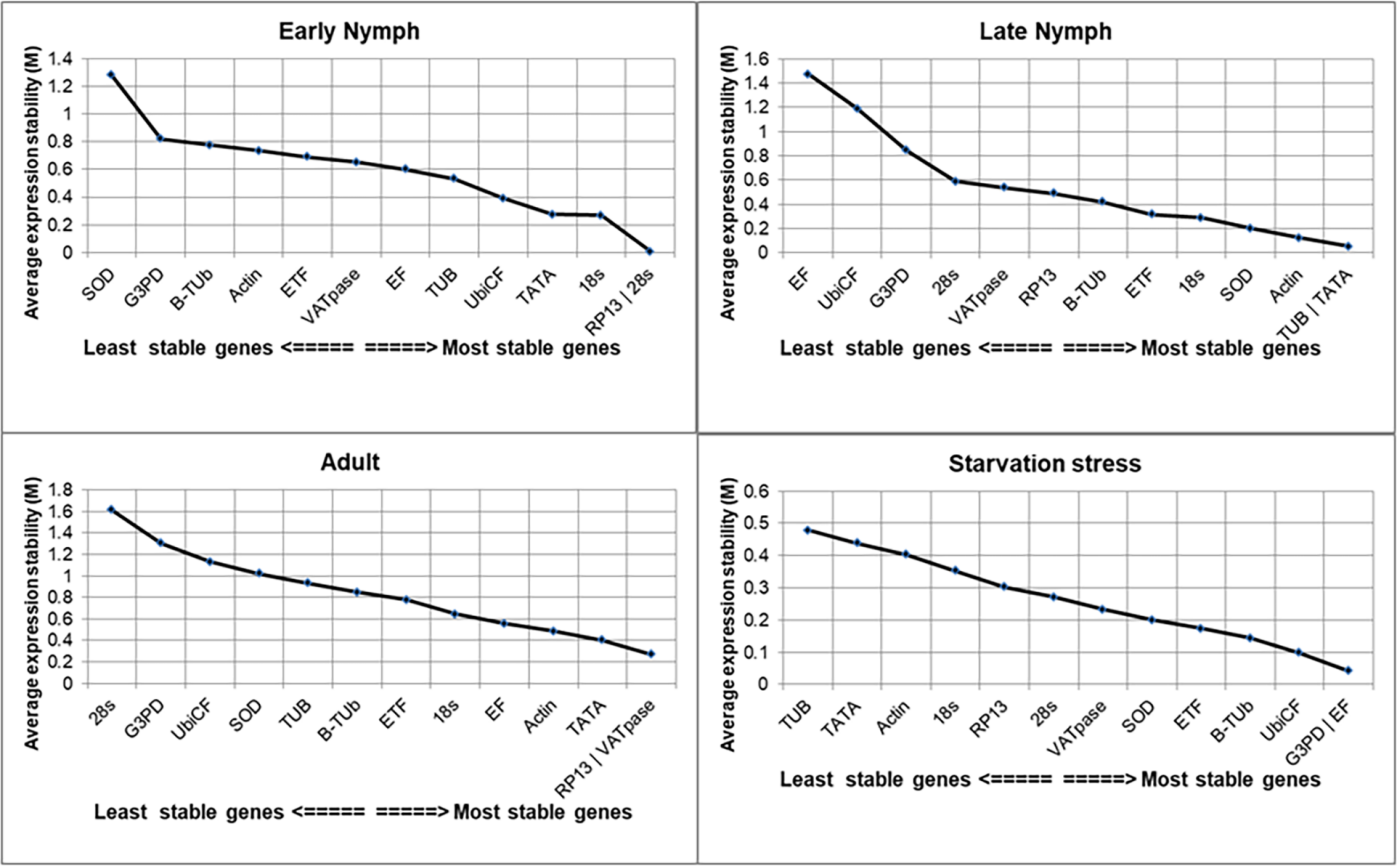
Average expression stability (M) of various in different developmental stages and starvation stress in A. *biguttula biguttula* analysis by geNorm.

Two parameters such as M (expression stability value) and V (pairwise variation) are determined by this program. The gene with the highest M value is considered least stable and vice versa. In early nymphal stage, RP13 and 28s were the most stable genes with M value of 0.005. Similarly, in late nymphs, Tub and TATA appeared as most stable genes with M value 0.05, while in adult hopper M value of 0.2 revealed RP13 and VATPase as the most stable genes. Under starvation stress GAPDH and EF were the most stable genes with M value is 0.04 (Fig 3).

### NormFinder analysis

**Fig 4:**
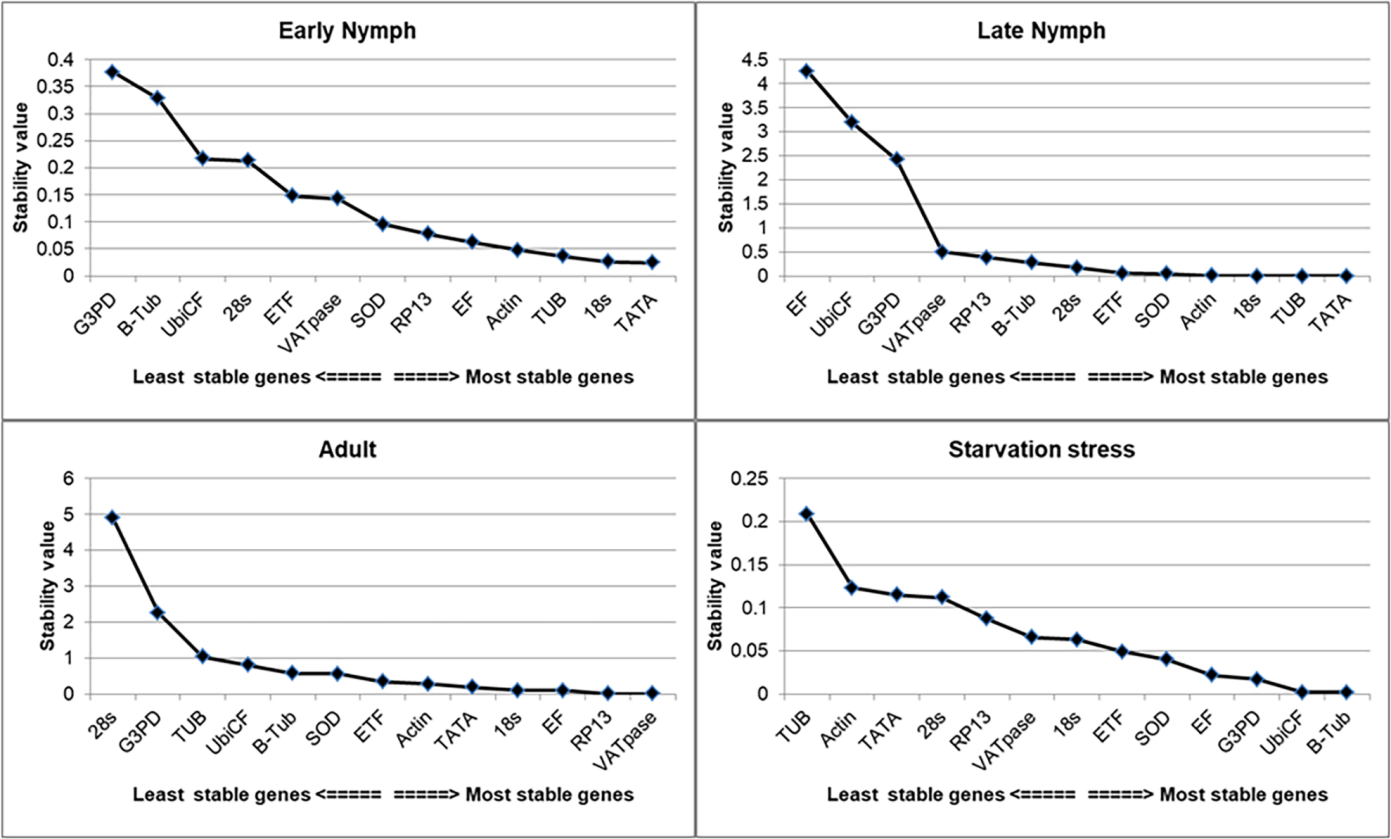
NormFinder analysis revealed stability values across the developmental stages and starvation stress.

Based on this program 18S was identified as best gene among all the samples showing stability value ~ 0.102. NormFinder also determines the best combination of two HKGs which can be used simultaneously in a single expression studies. Based on results, the combination of 18s and TATA was highly stable (stability value ~ 0.079). Under starvation stress, UbiCF appeared to be the most stable gene with a stability value of 0.034. The rankings of genes on the basis of their stability values are presented in Table 5 and graphically in Fig 4.

### RefFinder analysis

RefFinder assembles the output of all three algorithms described earlier along with ΔΔCt to assign final rankings for all the genes. It interprets the stability of genes by ranking of their geometric mean (Table 6), as well as allocates overall values to the genes across different stages (Table 7). These analyses showed that RP13, TUB, 28s and UbiCF were the most stable genes in the adult, late stage, early stage nymph and starvation stress, respectively. However, RefFinder also revealed RP13, VATPase, 18s in the adult; 28s, RP13, TUB in early nymph; TUB, TATA, Actin in late nymph and UbiCF, B-Tub, G3PD in starvation stress as the most stable genes. Based on overall analysis of all developmental stages, RP13 was identified as the best stable gene across all stages (Table 7). Thus, this gene was used as HKG for relative expression analysis in dsRNA feeding assays.

**Table 6:** Ranking of housekeeping genes in cotton leafhopper based on geometric mean using RefFinder software.

Adult		Late Nymph		Early Nymph		Starvation stress	
Genes	Geomean of ranking values	Genes	Geomean of ranking values	Genes	Geomean of ranking values	Genes	Geomean of ranking values
RP13	1.68	TUB	1.93	28s	1.86	UbiCF	2.06
VATpase	1.86	TATA	2.45	RP13	2.34	B-Tub	2.99
18s	2.91	Actin	2.63	TUB	2.91	G3PD	3.00
TATA	3.31	18s	3.87	UbiCF	3.76	EF	3.56
EF	5.1	SOD	4.47	EF	4.09	SOD	6.16
Actin	5.83	ETF	4.56	VATpase	5.42	TATA	6.31
B-TUb	6.45	28s	5.01	18s	6.19	ETF	6.65
ETF	7.00	B-Tub	7.48	TATA	7.28	18s	6.88
TUB	8.59	RP13	8.71	ETF	7.35	VATpase	8.10
SOD	9.21	VATpase	9.49	G3PD	10.04	Actin	8.12
UbiCF	10.49	G3PD	11.47	Actin	10.47	TUB	8.14
G3PD	12.00	UbiCF	11.74	B-Tub	10.93	RP13	8.45
28s	13.00	EF	12.74	SOD	13.00	28s	9.90

**Table 7:** The overall ranking of housekeeping genes in cotton leafhopper across different stages as well as starvation stress by Delta CT, BestKeeper, Normfinder and genorm algorithms.

**Adult**													
Method	1	2	3	4	5	6	7	8	9	10	11	12	13
Delta CT	RP13	VATpase	18s	TATA	EF	Actin	ETF	B-Tub	SOD	UbiCF	TUB	G3PD	28s
BestKeeper	18s	TATA	B-Tub	RP13	TUB	VATpase	ETF	Actin	EF	SOD	UbiCF	G3PD	28s
Normfinder	VATpase	RP13	EF	18s	TATA	Actin	ETF	SOD	B-TUb	UbiCF	TUB	G3PD	28s
Genorm	RP13 | VATpase		TATA	Actin	EF	18s	ETF	B-Tub	TUB	SOD	UbiCF	G3PD	28s
Recommended comprehensive ranking	**RP13**	**VATpase**	**18s**	**TATA**	**EF**	**Actin**	**B-Tub**	**ETF**	**TUB**	**SOD**	**UbiCF**	**G3PD**	**28s**
Late Instar													
Method	1	2	3	4	5	6	7	8	9	10	11	12	13
Delta CT	Actin	TUB	TATA	SOD	18s	ETF	B-Tub	RP13	28s	VATpase	G3PD	UbiCF	EF
BestKeeper	28s	ETF	18s	Actin	SOD	TATA	TUB	B-Tub	VATpase	RP13	UbiCF	EF	G3PD
Normfinder	TUB	TATA	18s	Actin	SOD	ETF	28s	B-Tub	RP13	VATpase	G3PD	UbiCF	EF
Genorm	TUB | TATA		Actin	SOD	18s	ETF	B-Tub	RP13	VATpase	28s	G3PD	UbiCF	EF
Recommended comprehensive ranking	**TUB**	**TATA**	**Actin**	**18s**	**SOD**	**ETF**	**28s**	**B-Tub**	**RP13**	**VATpase**	**G3PD**	**UbiCF**	**EF**
**Early Instar**													
Method	1	2	3	4	5	6	7	8	9	10	11	12	13
Delta CT	28s	RP13	TUB	EF	UbiCF	VATpase	18s	TATA	ETF	Actin	G3PD	B-TUb	SOD
BestKeeper	TUB	EF	VATpase	ETF	RP13	28s	G3PD	UbiCF	B-TUb	18s	TATA	Actin	SOD
Normfinder	UbiCF	28s	RP13	TUB	EF	VATpase	18s	TATA	ETF	Actin	G3PD	B-TUb	SOD
Genorm	RP13 | 28s		18s	TATA	UbiCF	TUB	EF	VATpase	ETF	Actin	B-TUb	G3PD	SOD
Recommended comprehensive ranking	**28s**	**RP13**	**TUB**	**UbiCF**	**EF**	**VATpase**	**18s**	**TATA**	**ETF**	**G3PD**	**Actin**	**B-TUb**	**SOD**
**Starvation Stress**													
Method	1	2	3	4	5	6	7	8	9	10	11	12	13
Delta CT	UbiCF	B-Tub	G3PD	EF	ETF	SOD	VATpase	18s	RP13	28s	Actin	TATA	TUB
BestKeeper	TATA	TUB	Actin	18s	B-Tub	UbiCF	RP13	SOD	G3PD	EF	VATpase	28s	ETF
Normfinder	UbiCF	B-Tub	G3PD	EF	SOD	ETF	18s	VATpase	RP13	28s	TATA	Actin	TUB
Genorm	G3PD | EF		UbiCF	B-Tub	ETF	SOD	VATpase	28s	RP13	18s	Actin	TATA	TUB
Recommended comprehensive ranking	**UbiCF**	**B-Tub**	**G3PD**	**EF**	**SOD**	**TATA**	**ETF**	**18s**	**VATpase**	**Actin**	**TUB**	**RP13**	**28s**
**Overal gene analysis**													
Method	**1**	**2**	**3**	**4**	**5**	**6**	**7**	**8**	**9**	**10**	**11**	**12**	**13**
Delta CT	**RP13**	**VATpase**	**TUB**	**ETF**	**TATA**	**18s**	**B-Tub**	**Actin**	**UbiCF**	**G3PD**	**EF**	**28s**	**SOD**
BestKeeper	**TUB**	**RP13**	**VATpase**	**ETF**	**B-Tub**	**18s**	**TATA**	**EF**	**UbiCF**	**Actin**	**G3PD**	**SOD**	**28s**
Normfinder	**RP13**	**VATpase**	**TUB**	**ETF**	**B-Tub**	**TATA**	**18s**	**Actin**	**UbiCF**	**G3PD**	**EF**	**28s**	**SOD**
Genorm	**RP13 | VATpase**		**TUB**	**B-TUb**	**ETF**	**TATA**	**18s**	**Actin**	**G3PD**	**UbiCF**	**EF**	**28s**	**SOD**
Recommended comprehensive ranking	**RP13**	**VATpase**	**TUB**	**ETF**	**B-Tub**	**TATA**	**18s**	**Actin**	**UbiCF**	**G3PD**	**EF**	**28s**	**SOD**

### Bioassay with gene-specific dsRNA:

**Fig 5:**
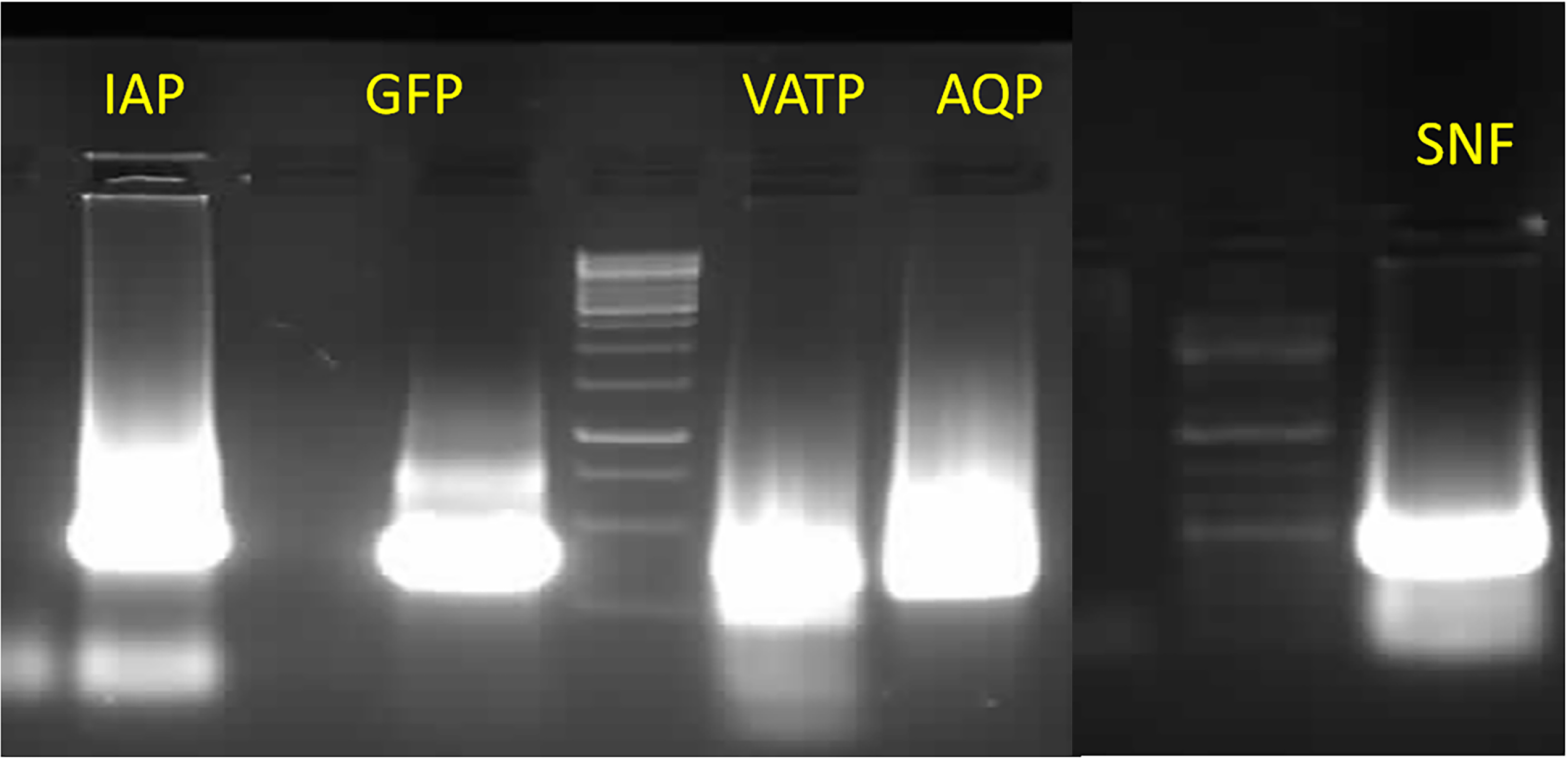
Visualization of different dsRNA on 1% agarose gel. The dsRNA was synthesized for RNAi studies in cotton leafhopper using diet bioassay.

**Fig 6:**
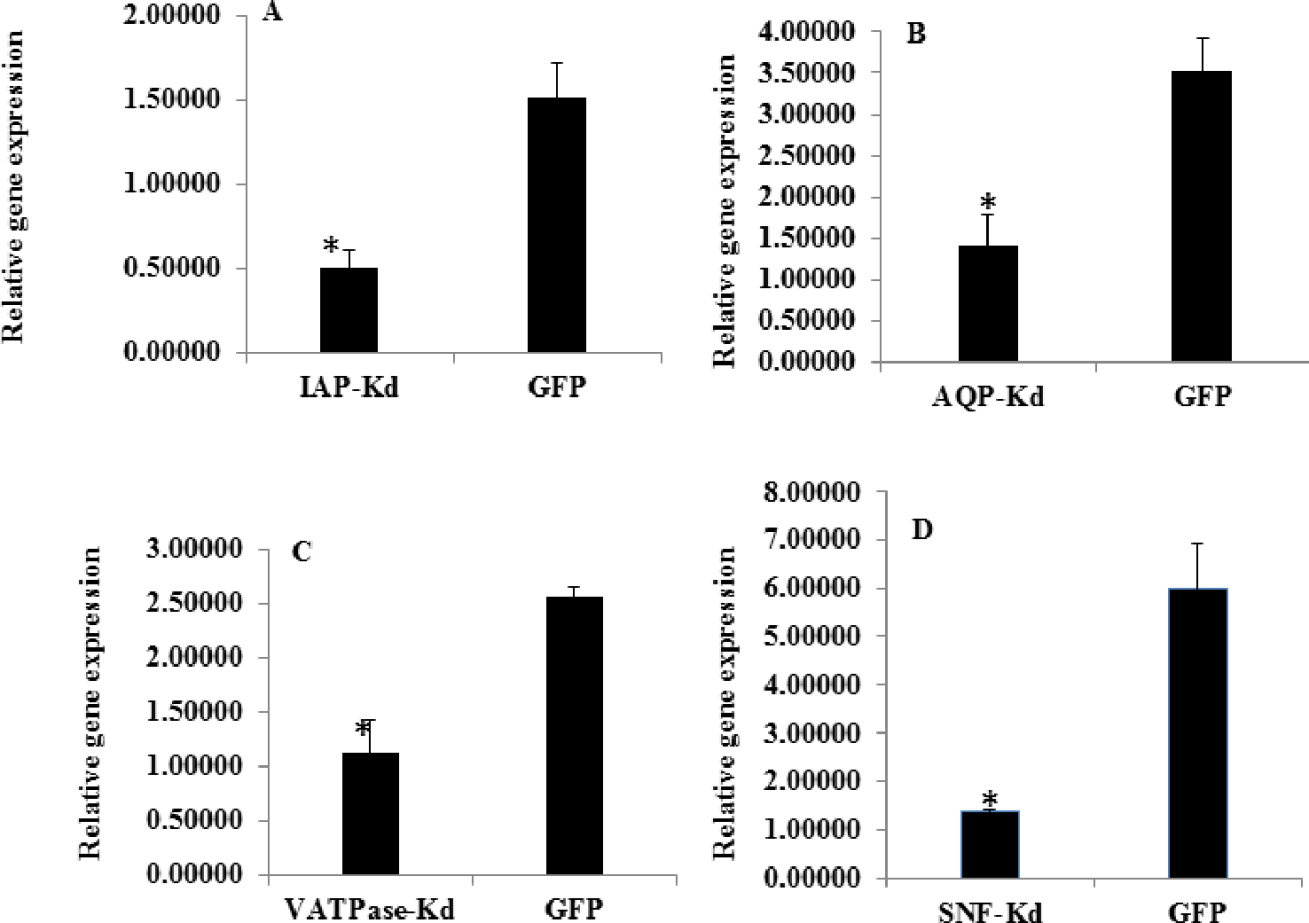
Expression of candidate genes in A. *biguttula biguttula* fed with dsRNA containing liquid diet by membrane feeding assay (A) Abb IAP (Inhibitor of Apoptosis) knockdown, (B) Abb AQP (Aquaporin) knockdown, (C) VATPase knockdown, (D) Snf7 (Multivesicular protein) knockdown. The mRNA levels of each gene have been normalized with ribosomal protein (RP13) as a reference gene. The error bars represent the standard deviation (n = 3) and * represents significant differences in mRNA levels compared to that in control—fed on dsGFP (P ≤ 0.05, Student’s t-test).

The target gene knockdown was confirmed by feeding the late stage nymphs with different dsRNAs using membrane feeding assay (Fig 5). Initial experiments using different doses of dsRNA showed that 500 ng/μl of dsRNA in diet caused knockdown of respective genes when compared to that in dsGFP fed control insects at 48 h post feeding. All the targeted genes showed a significant reduction (p≤ 0.05) in their mRNA levels compared to that in dsGFP fed control insects. The dsRNA feeding caused 4.37, 2.99, 2.49 and 2.28 fold reduction in the levels of Snf7, IAP, AQP and VATPase mRNA, respectively compared to that in control insects (Fig 6). The percent corrected mortality calculated using Abbott’s formula [41] varied between 16–48%; highest in dsSnf7 (48%) fed insects followed by dsAQP (27%), dsVATPase (20%) and dsIAP (16.7%) (Table 8); (S2 Table).

**Table 8:** Corrected percent mortality in the leafhopper nymphs at 48h after dsRNA feeding.

dsRNA Fed	Total no of test insects	% Corrected mortality after 48 h
Snf7	42	48.0
AQP	18	27.3
VATPase	42	20.0
IAP	18	16.7

## Discussion

In recent years, it has become clear that selection of an appropriate reference gene or genes is the basic requirement for the success of RT-qPCR [42]. However, in the most of the previously reported expression studies with insects, actin has been used as a universal HKG across species as well as across stages of insects [8,43]. Simultaneously, its use as an internal control has been contradictory in many cases [44,45]. Various studies have already been accomplished by identifying HKGs in different species and tissues. Recently, numerous reference genes have been testified under different conditions in stinkbug (Bansal *et al*, 2016), western flower thrips [46], honey bee [47], fly [48], silkworm [49], moths [50] and beetles [51]. The analysis of reference genes in cotton leafhopper across different developmental stages showed variation in expression as reported in several other insects [52]. These genes have significant involvement in ubiquitous cellular and biological processes, therefore cannot be used as single normalizer in expression studies. In order to improve the efficiency and accuracy of qRT-PCR, we need to validate various internal genes for their expression. RT-qPCR studies revealed variability in the expression of reference genes across samples inferring that a universal reference gene cannot be used for all species or all experimental conditions [53]. Thus validation of reference HKGs is obligatory to gene expression studies wherein a candidate reference gene should have amplification efficiency similar to the target gene.

In the current study, we evaluated fifteen candidate HKGs of cotton leafhopper using multiple statistical models (Bestkeeper, geNorm, NormFinder, and RefFinder) across different developmental stages and starvation stress for identification of reference genes as suggested by [54]. Interestingly, results obtained from different algorithms were highly variable. As each program is using the unique algorithm, incongruities are to be anticipated. For instance, the BestKeeper takes into account the InVar (intrinsic variance), SD (standard deviation) and *P*-values, all of which contribute to the *BestKeeper* vs. *Pearson* correlation coefficient value. These compounding factors result in the obvious differences in outcomes. To integrate the results of all three algorithms, we further used RefFinder for comprehensive ranking of the genes. RefFinder abdicated the ambiguity in the results and helped in the compilation of three algorithms as shown in Table 7.

Our results suggested that the RP13 gene was the most stable gene across all the developmental stages tested. The RP13 gene encodes a ribosomal protein that is a component of the 60S subunit, which is involved in the translation initiation [55]. Various ribosomal proteins genes have been evaluated as reference genes for RT-qPCR in many insects, and these genes have been reported to show the most stable expression in *Tetranychus cinnabarinus* (RPS18:[56]), *Apis mellifera* (RPS18: [47]), *Rhodnius prolixus* (RPS18: [57]), *Cimex lectularius* (RPL18: [58]), and *Schistocerca gregaria* (RP49: [59]). In late and early stage nymphs, Tub and 28s were the most stable genes, respectively. Previous studies also demonstrated 28s rRNA to be the stable and suitable gene for internal control in various organisms ([47, 60–63]. Tubulin has also been most commonly used as a reference gene in various expression studies [64–66]. These belong to the family of eukaryotic structural genes that form microtubules, fundamental components of the cytoskeleton that mediate cell division, shape, motility, and intracellular trafficking [67]. Nevertheless, tubulin is reported to be the most variable gene in plant hoppers [68]. Under starvation stress, UbiCF has come out to be the most stable gene in a comprehensive analysis. Ubiquitin is the founding member of a family of structurally conserved proteins that regulate a host of processes (protein degradation, DNA repair, signal transduction and transcription regulation by endocytosis) in insect cells [69–71]. Various genes coding for ubiquitin proteins such as ubiquitin conjugation factor (Bansal *et al*, 2016), polyubiquitin [68] and ubiquitin [64] evaluated in different insect species have shown stable expression as HKGs. Earlier reports concluded that it was difficult to identify most stable reference gene across various developmental stages in an organism [72–73]. We set our parameters under three algorithms (i.e. Bestkeeper, geNorm, Normfinder) to find the stable HKGs across developmental stages and starvation stress. However, the results obtained from individual algorithm were variable. Thus, we used RefFinder to comprehend the results of three algorithms and assign a ranking to the genes based on their stability under each and across (overall ranking) developmental stages and starvation stress. The overall ranking concludes RP13, VATPase and TUB as the most stable genes which could be helpful in the expression studies involving starvation stress and mixture of individuals from different life stages of leafhopper. Finally it may be concluded that top two-three genes could be selected and validated to elucidate the appropriate reference gene(s) under a particular set of experimental conditions for future gene expression studies in this insect.

Feeding of dsRNA for causing RNAi has been demonstrated in a few hemipteran insects including *Halyomorpha halys* [36]; *Bemisia tabaci* [74]; *Acyrthosiphon pisum* [75]; *Bactericera cockerelli* [76]; *Nilaparvata lugens* [77]; *Sogatella furcifera* [78]; *Laodelphax striatellus* [79]. Many insects in this order have also shown good RNAi response through injection of dsRNA [80–81]. In planta expressed dsRNA too has been successful in harnessing RNAi response in few of the hemipteran insects [82–87]. Gene silencing has been successfully demonstrated for many hemipteran insects through the oral delivery of synthetic dsRNAs dissolved in sucrose via artificial membrane [84,76,88]. The slightly similar and modified procedure has been used for delivering dsRNA in cotton leafhopper in the current study. Robust RNAi efficiency has been demonstrated in cotton leafhopper through the feeding of dsSnf7. Snf7 belongs to the ESCRT (Endosomal Sorting Complex Required for Transport)–III complex, the ESCRT pathway is a key regulator of biological processes important for eukaryotic cell growth and survival [89]. Feeding of dsRNA targeting western corn rootworm, Snf7 homolog in larvae caused severe stunting after five days of exposure followed by the death of the larvae [23]. The highest mortality was observed with the feeding of dsSnf7 to nymphs of cotton leafhopper. This may be correlated to 77.12% (4.3 fold) decline in the expression of the targeted gene compared to the GFP control. Aquaporin water channels have been implicated in mediating the mass transfer of water in a various physiological processes. Hemipteran water-specific aquaporins have been reported in the gut of phloem-feeding leafhopper *Cicadella viridis* [90] and *B*. *tabaci* [91]. The expression of the *Aquaporin 1* gene was reduced to 70% at six days post-feeding and caused 84% mortality in *B*. *tabaci* adults fed on 20 μg/ml A*QP1*dsRNA in 20% sucrose (Vyas *et al*, 2017). The reports showed no mortality in *A*. *pisum* fed on one μg/μl dsApAQP in the diet [92]. However, there was a two-fold reduction in the ApAQP expression compared to that in control. Thus, it is evident that the concentration of dsRNA might be a key factor for causing significant RNAi effect. In our experiments, dsRNA at 500 ng /μl diet caused 66.59% (2.99-fold) reduction in AbbAQP mRNA levels compared to that in dsGFP-fed control insects. However the percent corrected mortality caused by dsAbbAQP feeding was low compared with dsAbbSnf7 feeding. The mortality observed with the feeding of dsAbbIAP and dsAbbVATPase was also low. Nevertheless, there was a significant reduction of 59.87 and 56.17% in the mRNA levels of both the genes in the dsRNA fed insects. In some of the hemipteran insects, the downregulation of VATPase has resulted in a significant mortality as compared to the control [76,74,86,93]. RNAi mediated screening of 290 genes of western corn rootworm (*D*. *virgifera virgifera*) revealed that only 125 genes were able to cause larval mortality [94]. In lepidopteran insects, out of 130 genes, only 49 genes (38%) were silenced more than 50% whereas 18 genes (14%) showed lesser silencing while 62 (48%) genes did not show silencing at all [95]. In insects, *Hyalophora cecropia*, *Antheraea pernyi* and *Manduca sexta* even ten ng dsRNA per mg of insect biomass was sufficient to elicit high levels of target gene silencing [96–98]. Similarly, silencing of target genes in coleopteran beetles could be achieved with small quantities of dsRNA [99]. Contrastingly, very high concentration (100 μg) of dsRNA per mg of insect biomass was required to silence target gene of *Antheraea mylitta* [100].The length of dsRNA is also one of the critical parameters that determine dsRNA uptake and RNAi efficiency [101–102]. To conclude, the RNAi efficiency in cotton leafhopper with different genes showed a differential response. Thus exhaustive studies in future may be helpful to better understand the RNAi and its core machinery in this insect. Overall, the current study has identified stable reference genes across various developmental stages and starvation stress in *A*. *biguttula biguttula*. Considered in concert, using different software algorithms and comprehensive analysis results, we suggest that RP13 and VATPase are the most suitable HKGs for gene expression studies across developmental stages. RefFinder concluded RP13, VATPase, 18s in adult, 28s, RP13, TUB in early stage nymph, TUB, TATA, Actin in late- stage nymph and UbiCF, B-Tub, G3PD in starvation stress as the top three stables genes. However, considering the individual developmental stage RP13, Tub and 28S are best suited for adult, late, and early nymphal stages, respectively. In addition, UBiCF is most stable HKG under starvation stress. This study also reports for the first time successful gene silencing through RNAi in *A*. *biguttula biguttula*.

## Supporting information

S1 Table Descriptive analysis of all candidate reference genes by calculating standard deviation and p-values by Bestkeeper. (DOC)

S2 Table Mortality calculated in bioassays with dsRNA of different genes in comparison to dsGFP. (DOCX)
