# Supplementary material for: Selection of housekeeping genes and demonstration of RNAi in cotton leafhopper, Amrasca biguttula biguttula (Ishida)
Source: PLoS One. 2018 Jan 12;13(1):e0191116. doi: 10.1371/journal.pone.0191116 (PMC5766320; doi:10.1371/journal.pone.0191116)
Supplement: S1 Table — (DOC) [file pone.0191116.s001.doc]

S1 Table: Descriptive Analysis of all candidate reference genes by calculating standard deviation and p-values by Bestkeeper

| Adult |  |  |  |  |  |  |  |  |  |  |  |  |  |
| --- | --- | --- | --- | --- | --- | --- | --- | --- | --- | --- | --- | --- | --- |
|  | UbiCF | G3PD | RP13 | VATpase | TUB | B-TUb | EF | Actin | 18s | 28s | TATA | ETF | SOD |
| N | 3 | 3 | 3 | 3 | 3 | 3 | 3 | 3 | 3 | 3 | 3 | 3 | 3 |
| geo Mean [CP] | 27.05 | 24.2 | 22.74 | 23.53 | 26.25 | 22.95 | 24 | 28.11 | 26.83 | 26.79 | 26.98 | 24.14 | 28.06 |
| AR Mean [CP] | 27.1 | 24.28 | 22.75 | 23.54 | 26.26 | 22.96 | 24.02 | 28.13 | 26.83 | 26.96 | 26.98 | 24.15 | 28.09 |
| min [CP] | 25.16 | 21.66 | 22.26 | 22.78 | 25.78 | 22.3 | 22.87 | 26.93 | 26.33 | 23.39 | 26.37 | 23.13 | 26.4 |
| max [CP] | 29.06 | 25.75 | 23.44 | 24.5 | 27.02 | 23.58 | 25.44 | 28.94 | 27.29 | 30.79 | 27.35 | 24.69 | 29.48 |
| stddev [+/- CP] | 1.31 | 1.74 | 0.46 | 0.64 | 0.51 | 0.44 | 0.94 | 0.8 | 0.34 | 2.55 | 0.41 | 0.68 | 1.13 |
| CV [% CP] | 4.83 | 7.19 | 2.02 | 2.73 | 1.94 | 1.91 | 3.93 | 2.84 | 1.25 | 9.47 | 1.52 | 2.81 | 4.01 |
| min [x-fold] | -3.71 | -5.83 | -1.4 | -1.68 | -1.39 | -1.57 | -2.19 | -2.27 | -1.41 | -10.56 | -1.53 | -2.01 | -3.16 |
| max [x-fold] | 4.03 | 2.92 | 1.62 | 1.96 | 1.7 | 1.55 | 2.71 | 1.77 | 1.38 | 15.99 | 1.29 | 1.47 | 2.67 |
| stddev [+/- x-fold] | 2.48 | 3.35 | 1.38 | 1.56 | 1.42 | 1.35 | 1.92 | 1.74 | 1.26 | 5.87 | 1.33 | 1.6 | 2.18 |
| Pearson correlation coeff |  |  |  |  |  |  |  |  |  |  |  |  |  |
| BestKeeper vs. | UbiCF | G3PD | RP13 | VATpase | TUB | B-TUb | EF | Actin | 18s | 28s | TATA | ETF | SOD |
| coeff. of corr. [r] | 0.886 | 0.462 | 0.978 | 0.959 | 0.001 | 0.015 | 0.95 | 0.687 | 0.804 | 0.881 | 0.618 | 0.509 | 0.752 |
| p-value | 0.045 | 0.433 | 0.004 | 0.01 | 0.244 | 0.98 | 0.013 | 0.2 | 0.101 | 0.048 | 0.267 | 0.381 | 0.143 |
|  |  |  |  |  |  |  |  |  |  |  |  |  |  |
| Late Instar |  |  |  |  |  |  |  |  |  |  |  |  |  |
| N | 3 | 3 | 3 | 3 | 3 | 3 | 3 | 3 | 3 | 3 | 3 | 3 | 3 |
| geo Mean [CP] | 30.48 | 26.12 | 23.6 | 24.02 | 26.15 | 22.18 | 27.93 | 26 | 25.28 | 22.82 | 26.07 | 22.43 | 27.19 |
| AR Mean [CP] | 30.54 | 26.22 | 23.63 | 24.05 | 26.16 | 22.2 | 28.01 | 26.01 | 25.28 | 22.82 | 26.08 | 22.44 | 27.2 |
| min [CP] | 28.73 | 24.57 | 22.49 | 22.74 | 25.65 | 21.16 | 26.23 | 25.57 | 25.03 | 22.67 | 25.53 | 22.08 | 26.54 |
| max [CP] | 33.13 | 29.44 | 25.24 | 25.61 | 27.16 | 23.66 | 30.99 | 26.82 | 25.74 | 22.96 | 27.07 | 22.89 | 28.05 |
| stddev [+/- CP] | 1.73 | 2.15 | 1.08 | 1.04 | 0.67 | 0.97 | 1.99 | 0.54 | 0.3 | 0.1 | 0.66 | 0.3 | 0.57 |
| CV [% CP] | 5.66 | 8.2 | 4.55 | 4.33 | 2.55 | 4.37 | 7.1 | 2.08 | 1.2 | 0.44 | 2.52 | 1.35 | 2.09 |
| min [x-fold] | -3.36 | -2.93 | -2.16 | -2.42 | -1.41 | -2.03 | -3.25 | -1.35 | -1.19 | -1.11 | -1.46 | -1.28 | -1.57 |
| max [x-fold] | 6.28 | 9.98 | 3.12 | 3.02 | 2.01 | 2.79 | 8.35 | 1.76 | 1.37 | 1.1 | 1.99 | 1.37 | 1.82 |
| stddev [+/- x-fold] | 3.31 | 4.43 | 2.11 | 2.06 | 1.59 | 1.96 | 3.97 | 1.46 | 1.23 | 1.07 | 1.58 | 1.23 | 1.48 |
| Pearson correlation coeff |  |  |  |  |  |  |  |  |  |  |  |  |  |
| BestKeeper vs. | UbiCF | G3PD | RP13 | VATpase | TUB | B-TUb | EF | Actin | 18s | 28s | TATA | ETF | SOD |
| coeff. of corr. [r] | 0.001 | 0.976 | 0.894 | 0.823 | 0.975 | 0.888 | 0.001 | 0.982 | 0.985 | 0.684 | 0.987 | 0.851 | 0.86 |
| p-value | 0.915 | 0.005 | 0.041 | 0.087 | 0.005 | 0.044 | 0.773 | 0.003 | 0.002 | 0.202 | 0.002 | 0.068 | 0.062 |
| Early Instar |  |  |  |  |  |  |  |  |  |  |  |  |  |
| N | 3 | 3 | 3 | 3 | 3 | 3 | 3 | 3 | 3 | 3 | 3 | 3 | 3 |
| geo Mean [CP] | 28.47 | 27 | 22.9 | 23.73 | 25.06 | 23.19 | 26.22 | 24.34 | 23.91 | 22.94 | 24.27 | 23.21 | 30.2 |
| AR Mean [CP] | 28.48 | 27.01 | 22.9 | 23.74 | 25.06 | 23.21 | 26.22 | 24.36 | 23.92 | 22.94 | 24.28 | 23.21 | 30.36 |
| min [CP] | 27.93 | 26.4 | 22.28 | 23.43 | 24.94 | 22.45 | 26.09 | 23.06 | 22.84 | 22.32 | 23.19 | 22.8 | 26.68 |
| max [CP] | 29.19 | 27.7 | 23.24 | 24.12 | 25.18 | 24.27 | 26.45 | 25.24 | 24.5 | 23.28 | 24.85 | 23.73 | 34.32 |
| stddev [+/- CP] | 0.48 | 0.46 | 0.41 | 0.26 | 0.08 | 0.71 | 0.15 | 0.86 | 0.72 | 0.42 | 0.73 | 0.35 | 2.64 |
| CV [% CP] | 1.67 | 1.71 | 1.8 | 1.08 | 0.33 | 3.05 | 0.58 | 3.55 | 3.01 | 1.81 | 3 | 1.49 | 8.69 |
| min [x-fold] | -1.46 | -1.52 | -1.53 | -1.24 | -1.08 | -1.67 | -1.1 | -2.43 | -2.1 | -1.54 | -2.12 | -1.33 | -11.5 |
| max [x-fold] | 1.65 | 1.62 | 1.27 | 1.31 | 1.09 | 2.11 | 1.17 | 1.87 | 1.51 | 1.27 | 1.49 | 1.44 | 17.35 |
| stddev [+/- x-fold] | 1.39 | 1.38 | 1.33 | 1.19 | 1.06 | 1.63 | 1.11 | 1.82 | 1.65 | 1.33 | 1.66 | 1.27 | 6.22 |
| Pearson correlation coeff |  |  |  |  |  |  |  |  |  |  |  |  |  |
| BestKeeper vs. | UbiCF | G3PD | RP13 | VATpase | TUB | B-TUb | EF | Actin | 18s | 28s | TATA | ETF | SOD |
| coeff. of corr. [r] | 1 | 0.001 | 0.697 | 0.001 | 0.001 | 0.019 | 0.001 | 0.859 | 0.706 | 0.703 | 0.721 | 0.001 | 0.722 |
| p-value | 0.001 | 0.498 | 0.191 | 0.421 | 0.571 | 0.976 | 0.223 | 0.062 | 0.183 | 0.185 | 0.169 | 0.409 | 0.169 |
| Starvation stress |  |  |  |  |  |  |  |  |  |  |  |  |  |
| N | 3 | 3 | 3 | 3 | 3 | 3 | 3 | 3 | 3 | 3 | 3 | 3 | 3 |
| geo Mean [CP] | 29.8 | 26.74 | 24.7 | 24.92 | 26.95 | 22.66 | 23.99 | 25.97 | 25.23 | 23.08 | 25.82 | 22.24 | 26.02 |
| AR Mean [CP] | 29.8 | 26.75 | 24.71 | 24.93 | 26.95 | 22.66 | 23.99 | 25.97 | 25.23 | 23.09 | 25.82 | 22.25 | 26.02 |
| min [CP] | 29.46 | 26.37 | 24.1 | 24.33 | 26.72 | 22.38 | 23.57 | 25.55 | 24.78 | 22.37 | 25.73 | 21.77 | 25.45 |
| max [CP] | 30.34 | 27.42 | 25.16 | 25.64 | 27.37 | 23.13 | 24.68 | 26.32 | 25.47 | 23.8 | 25.88 | 23.02 | 26.67 |
| stddev [+/- CP] | 0.36 | 0.45 | 0.4 | 0.48 | 0.28 | 0.31 | 0.46 | 0.28 | 0.3 | 0.48 | 0.06 | 0.51 | 0.43 |
| CV [% CP] | 1.2 | 1.68 | 1.64 | 1.91 | 1.03 | 1.37 | 1.91 | 1.09 | 1.19 | 2.08 | 0.24 | 2.31 | 1.67 |
| min [x-fold] | -1.27 | -1.29 | -1.52 | -1.51 | -1.17 | -1.21 | -1.34 | -1.34 | -1.36 | -1.64 | -1.07 | -1.39 | -1.48 |
| max [x-fold] | 1.45 | 1.6 | 1.37 | 1.65 | 1.34 | 1.38 | 1.62 | 1.27 | 1.18 | 1.64 | 1.04 | 1.71 | 1.57 |
| stddev [+/- x-fold] | 1.28 | 1.36 | 1.32 | 1.39 | 1.21 | 1.24 | 1.37 | 1.22 | 1.23 | 1.39 | 1.04 | 1.43 | 1.35 |
| Pearson correlation coeff |  |  |  |  |  |  |  |  |  |  |  |  |  |
| BestKeeper vs. | UbiCF | G3PD | RP13 | VATpase | TUB | B-TUb | EF | Actin | 18s | 28s | TATA | ETF | SOD |
| coeff. of corr. [r] | 0.993 | 1 | 0.688 | 0.916 | 0.001 | 0.986 | 0.996 | 0.212 | 0.569 | 0.837 | 0.001 | 0.983 | 0.933 |
| p-value | 0.001 | 0.001 | 0.199 | 0.029 | 0.505 | 0.002 | 0.001 | 0.732 | 0.317 | 0.077 | 0.001 | 0.003 | 0.021 |

|  |  |  |  |  |  |  |  |  |  |
| --- | --- | --- | --- | --- | --- | --- | --- | --- | --- |
|  |  |  |  |  |  |  |  |  |  |
|  |  |  |  |  |  |  |  |  |  |
|  |  |  |  |  |  |  |  |  |  |
|  |  |  |  |  |  |  |  |  |  |
