# Supplementary material for: Selection of housekeeping genes and demonstration of RNAi in cotton leafhopper, Amrasca biguttula biguttula (Ishida)
Source: PLoS One. 2018 Jan 12;13(1):e0191116. doi: 10.1371/journal.pone.0191116 (PMC5766320; doi:10.1371/journal.pone.0191116)
Supplement: S2 Table — (DOCX) [file pone.0191116.s002.docx]

S2 Table: Mortality calculated in bioassays with dsRNA of different genes in comparison to dsGFP.

| **S.No.** | **dsRNA** | **No. of test insects** | **Live insects after 48 hrs**  **(T)** | **% Mortality** | **dsRNA** | **No. of test insects** | **Live insects after 48 hrs**  **(C)** | **% Mortality** | **Corrected % Mortality** |
| --- | --- | --- | --- | --- | --- | --- | --- | --- | --- |
| 1 | SNF | 42 | 13 | 69.05 | GFP | 42 | 25 | 40.48 | 48.0 |
| 2 | AQP | 18 | 8 | 55.56 | GFP | 18 | 11 | 38.89 | 27.3 |
| 3 | VATPase | 42 | 20 | 52.38 | GFP | 42 | 25 | 40.48 | 20.0 |
| 4 | IAP | 18 | 10 | 44.44 | GFP | 18 | 12 | 33.33 | 16.7 |
